# Economic Impacts of Treatment for Type II or III Thoracoabdominal Aortic Aneurysm in the United States

**DOI:** 10.5812/cardiovascmed.9568

**Published:** 2014-02-24

**Authors:** Mickael Vaislic, Claude Vaislic, Jean-Marc Alsac, Amira Benjelloun, Sidney Chocron, Thierry Unterseeh, Jean-Noel Fabiani

**Affiliations:** 1 European Company of Strategic Intelligence, Paris, France; 2 Cardiovascular Surgery, Parly II Private Hospital, Le Chesnay, France; 3 Vascular Surgery, Georges Pompidou European Hospital, Paris, France; 4 Vascular Surgery, Medical and Surgical Centre for Heart and Vessels, Sale, Morocco; 5 Cardiac Surgery, University Hospital, Besancon, France; 6 Cardiology Department, Claude Galien Private Hospital, Quincy-sous-Senart, France; 7 Cardiac Surgery, Georges Pompidou European Hospital, Paris, France

**Keywords:** Aneurysm, Ruptured, Aortic Aneurysm, Thoracic, Endovascular Procedures, Cardiovascular Surgical Procedures, Paraplegia, Health Care Sector

## Abstract

**Background:**

Current treatment for extensive thoracoabdominal aortic aneurysms (TAAAs) involves high-risk surgical and endovascular repairs, with a hospital mortality exceeding 20%, and a postoperative paraplegia rate beyond 10.5%.

**Objectives:**

The aim of this study was to present an estimation of the economic impacts of surgical and endovascular treatments of types II and III TAAAs in the US as well as the economic consequences of the elimination of spinal cord injury and mortality via an endovascular repair of extensive TAAAs (1).

**Materials and Methods:**

We compared the current hospital charges of endovascular and surgical repair of extensive TAAAs, also provided a cost analysis of health care charges resulting from paraplegia in the United States, and determined the prevalence of extensive TAAAs found yearly during autopsies in the U.S. Based on the figures gathered and the frequency of Thoracic Aortic Aneurysms per year, we were able to calculate the nationwide inpatient hospital charges, the total average expenses affected by paraplegia during the first 12 months after the repair, the total average expenses after paraplegia for each subsequent year, mortality rate at 30 days and one year, and the number of extensive TAAAs ruptures.

**Results:**

The current nationwide inpatient hospital charges for type II or III TAAA repair cost $12484324 and $37612665 for endovascular repair and surgical repair respectively, and the total average expenses for patients affected by paraplegia during the first 12-month were $4882291 and $23179110 after endovascular repair and surgical repair respectively. The nationwide average expense after 10 years for patients undergoing surgical repair and affected by paraplegia is $33421910 and $6,316,183 for patients undergoing endovascular repair. Moreover, 55 patients with a type II or type III TAAA died after 30 days, and 100 after 1 year. The potential risk of type II or III TAAA ruptures is totally 1637 in a year.

**Conclusions:**

Major economic impacts of type II or III TAAA repairs in the United States have been identified. An endovascular repair excluding spinal cord injury and mortality with the same average costs as present endovascular treatments makes it possible to save at least $53189742 after one year, 100 lives of operated patients would be saved after one year, and 1637 type II and III TAAA ruptures would be avoided yearly.

## 1. Background

Surgical treatments for aortic aneurysms have made a significant progress in the past decade, with a marked fall in both morbidity and mortality. The major remaining problems concerning therapy for extensive thoracoabdominal aortic aneurysms (TAAAs), which current treatment involves high-risk surgery in a frail, elderly population, include the potential risk of postoperative paraplegia. Although acceptable rates of adverse outcome for TAAA resection at aortic surgery centers in highly selected patients have been reported, nationwide estimates of hospital mortality exceed 20%. In consequence of the high morbidity of open surgical repair, most patients with TAAA are currently never referred for elective treatment, and the results of emergency surgery are so dismal that some centers refuse to undertake these operations in elderly patients. Thus the possibility of treatment of extensive TAAA by endovascular techniques has enormous potential benefits. But before endovascular therapy for extensive TAAA can become a reality, more reliable spinal cord protection must be guaranteed. Indeed, endovascular repair of these aneurysms involves a risk of paraplegia as high as, or higher than, open surgical treatment by experienced surgeons.

## 2. Objectives

This study presents a comparison between the current hospital charges resulting from endovascular and surgical repair of types II and III TAAAs, and the consequences - i.e. the estimation of the savings related to nationwide average expenses for patients affected by paraplegia, the number of lives saved, and the number of type II or III TAAA ruptures avoided - of the exclusion of spinal cord injury and mortality via an endovascular repair. 

## 3. Materials and Methods

Our methodology was based on four principles.

### 3.1. Determination of Mortality Rates and Occurrences of Spinal Cord Injury After Endovascular and Surgical Repairs of Types II and III TAAAs

Many series reported a relatively low risk of death and paraplegia after Endovascular Repair (ER), ranging from 1.6% to ~3% (2, 3). However, these studies were performed on selected patient populations with disease limited to the thoracic aorta. Recently, some alternatives to conventional open surgery have been described for extensive aneurysms involving the thoracoabdominal segments (types II and III). These strategies include the use of extra-anatomic bypass followed by total aortic relining with stent grafts, as well as endovascular repair with branched grafts (4). A comparison between the efficiency of endovascular and surgical repairs of extensive TAAAs is therefore relevant. In 2008, Greenberg et al. (5) presented a study on 724 patients (352 ER and 372 surgical repairs) treated between January 1st, 2001 and July 31st, 2006 at the Cleveland Clinic Foundation in Cleveland, Ohio who underwent elective open surgical repair (SR) or ER of descending thoracic or thoracoabdominal aneurysms. Patients with aortic rupture or acute dissection and others who underwent emergent operation were excluded. No intraoperative deaths occurred in the series. The mean age was 67 ± 12 years; 65% of the patients were male. The mortality rate for these patients with types II and III thoracoabdominal aneurysms by repair technique and the proportion of patients who developed spinal cord injury (SCI) are excerpted in Table 1.

**Table 1.** Mortality and Spinal Cord Injury for Patients With Type II and III Aneurysmal Disease (6)

Extent	Repair	Patients, No. (%)	Mortality^a^at 30 days, No. (%)/Rate^b^	Mortality^a^at 1 year, No. (%)/Rate^b^	Spinal Cord Injury, No. (%)
**Type II**	ER^c^	16 (5)	1 (6)/0.80	5 (36)/0.45	3 (19)
	SR^c^	59 (16)	10 (17)/2.36	13 (22)/0.30	13 (22)
**Type III**	ER	22 (6)	2 (9)/1.16	7 (34)/0.52	1 (5)
	SR	62 (17)	8 (12)/1.68	13 (21)/0.27	6 (10)
**Type II and III**	ER	38 (10.8)	3 (7.9)	12 (31.6)	4 (10.5)
	SR	121 (32.5)	18 (14.9)	26 (21.5)	19 (15.7)

^a^ Mortality rates are expressed as Kaplan-Meier estimates determined from life-table analyses and cases per person/year.

^b^ Incidence rates are defined as deaths per person/year.

^c^ Abbreviations: ER, endovascular repair; SR, surgical repair.

Notably, the 30-day mortality rate was higher for patients with types II and III aneurismal disease undergoing surgical repair (type II: 17% for SR vs. 6% for ER; type III: 12% for SR vs. 9% for ER). The trend is however reversed when one-year mortality rates are considered (type II: 22% for SR vs. 36% for ER; type III: 21% for SR vs. 34% for ER). The occurrence of spinal cord injury was highest in both repair techniques for type II aneurysms (type II: 22% for SR vs. 19% for ER; type III: 10% for SR vs. 5% for ER). The severity of the SCI (paraplegia vs. paraparesis) and the potential for recovery did not differ between the treatment modalities. Therefore, interventions (endovascular or surgical repair) for a TAAA appear to have relatively high risks of mortality and morbidity, including the risk of spinal cord ischemic injury (6). An article written by Conrad et al. (7) provided a 20-year perspective of TAAA surgical repairs and confirmed the relatively high risk of mortality and SCI pertaining to this technique described in Greenberg’s study (5). The 30-day mortality rate for patients with types II and III thoracoabdominal aneurysms, and the proportion of patients who developed immediate or delayed Spinal Cord Injury (SCI) are shown in Table 2.

**Table 2.** Mortality and Spinal Cord Injury Classified by Extent of Aneurysmal Disease^a^ (8)

Extent	Patients, No. (%)	Mortality, No. (%), 30 days	Spinal Cord Injuries Overall, No. (%)	Spinal Cord Injuries Overall, %	
				Immediate	Delayed
**Type II**	69 (15.2)	6 (8.7)	14 (20.3)	17.4	2.9
**Type III**	164 (36)	17 (10.3)	24 (14.6)	11.6	3.0
**Type II and III**	233 (51.2)	23 (9.9)	38 (16.3)	13.3	3.0

^a^ Spinal cord injuries were classified as immediate when noted as the patient awoke from anesthesia or delayed when patients were initially neurologically intact.

Conrad et al. observed an operative mortality of 9.87% and a one-year mortality rate of 20% for patients with a mean age of 71.1 years with types II and III TAAAs. The SCI occurrence was 16.31% for these patients.

### 3.2. Determination of the Inpatient Hospital Charges for a TAAA Repair

Thanks to a panel of experts (9), we established a description of the costs parameters that should be taken into account to compare SR and ER treatment approaches of TAAAs (Table 3).

**Table 3.** Parameters Used to Compute Hospital Charges Pertaining to Surgical and Endovascular Repair of TAAAs

Surgical Repair	Endovascular Repair
**Diagnosis Phase**	
Cost of a thoracic radiography	
Cost of a transthoracic Doppler echocardiography	
Cost of a scan	
Cost of an angiography with a graduated-marker catheter	
Cost of a magnetic resonance angiography	
Cost of a specialized surgeon consultation	
**Pretherapeutic check-up**	
Cost of a biochemical profile: CBC, electrolytes, urea, creatinine, fibrin, PTT^a^	
Cost of a functional respiratory exploration, arterial gasometry	
Doppler echocardiography of the neck vessels (treatment of a tight aortic stenosis of the internal carotid artery before the aneurysm treatment if necessary)	
Cost of cardiac examinations: echocardiography, coronarography (if surgery is considered)	
Cost of an anesthesia consultation	
Cost of cardiology consultation	
**Hospitalization (check-up not included)**	
Cost of hospitalization (function of the number of average hospitalization days)	
**Intervention**	
Cost of the operating room occupation	
Cost of the length of intervention (function of the average number of hours)	
**Transfusion cost**	
Cost of instrumentation according to small and big devices/single-use or multiple-use	
Cost of radiologic angiographic device	
Cost of the prosthesis placed	Cost of the endoprosthesis placed
Cost of personnel: 2 surgeons, 1 anesthesiologist, 1 surgical assistant, 1 dresser, 1 instrumentist, 1 PA^a^	Cost of personnel: surgeon, 1 anesthesiologist, 1 radiologist, 1 X-ray technician
**Postoperative follow-up**	
Cost of a stay in ICU^a^	Cost of a stay in ICU
**Supervision**	
Cost of consultations after 1 month, 1 year, then each year	Cost of consultations and radiological explorations after 1, 3 (if there is a residual endoleak during postoperative assessment) and 6 months, then each year
Cost of a thoracic and abdominal scan	Cost of a thoracic and abdominal scan
NA^a^	Cost of a thoracic radiography

^a^ Abbreviations: PA, Physician Assistant; ICU, intensive care unit; NA, not available; PTT, partial thromboplastin time.

These parameters reflect an example of the United States’ practices, and pinpoint the elements significantly influencing hospital charges. The calculations details of the inpatient hospital charges for a TAAA repair are provided in section 5.

### 3.3. Determination of the Average Expenses per Patient After Paraplegia for the First Year and for Each Subsequent Year

Charges for rehospitalizations include all of the types of charges listed previously for inpatient acute care and rehabilitation, as well as charges for emergency room visits when no hospitalization occurred. The cost of each day hospitalized was based on the results of the study by DeVivo and Farris (15) entitled Topics in Spinal Cord Injury Rehabilitation, volume 16, Number 4. Briefly, 430 rehospitalizations were identified among patients whose initial rehabilitation occurred at the University of Alabama at Birmingham (UAB). Billing records were obtained from each hospital as well as Alabama Medicaid computer listings when necessary. Approximately 50% of the rehospitalizations occurred at UAB, and the remaining occurred at other hospitals. Nursing home care days are based on self-report of all persons in the National Spinal Cord Injury Statistical Center (NSCISC) database who completed an annual follow-up evaluation between 2000 and 2006, and had complete data on this item (n = 8239). The cost of each day in a nursing home was based on the national average cost of a semiprivate room in 2009 ($198 per day) given by the MetLife Mature Market Institute. The charges include physician fees associated with nursing home visits. Outpatient Services include physical therapy (even when conducted in the patient’s home), occupational therapy, speech therapy, psychology, laboratory, X-ray, and other miscellaneous charges associated with outpatient visits. Charges for time spent in transitional living units are also included. Physician visits and associated charges are considered separately.

Durable equipment charges include all charges for purchase, rental, or repair of equipment such as hospital beds, special mattresses, commodes, wheelchairs, wheelchair cushions, wheelchair backpacks, cushion covers, caliper straps, crutches, braces, splints, orthoses, ventilators, ventilator parts, and environmental control units. The purchase or lease price of a new car or van was specifically excluded, since individuals in the general population presumably purchase new cars at the same pace as persons with SCI. Environmental charges include all charges for modifications to residences and work sites. The purchase or lease price of a new car or van was once again specifically excluded. However, if a car or van already owned or newly purchased by an individual is appropriately modified during the year (hand controls, lift, etc.), then charges for these modifications were included. Charges for medications and supplies include those of both prescription and nonprescription drugs and supplies. Typical medications included muscle relaxers, urine acidifiers, antacids, laxatives, analgesics, and antibiotics. Typical supplies included catheters, tubing leg and bed bags, disposable gloves, adhesive tape, cement, detergent, skin lotions, powder, bed pads, bandages, and diapers.

Charges for attendant care are based on the national average cost of a home health aide in 2009 ($21 per hour) given by the MetLife Mature Market Institute. This estimate was based on a national survey of home care agencies and reflected their private pay rate rather than their Medicare or Medicaid reimbursed rate. Attendant care hours per day were based on persons self-report in the National Spinal Cord Injury Statistical Center (NSCISC) database who completed an annual follow-up evaluation between 2000 and 2006, and had complete data on this item (n = 7637). The charges include those for unskilled services provided by paid family members, friends, neighbors, aides, and orderlies, as well as skilled services provided by registered nurses or licensed practical nurses. They include both paid and unpaid services. Vocational rehabilitation charges include all charges for vocational and educational preparation and vocational counseling purchased either by the person with SCI or a third party (such as a vocational rehabilitation agency). Miscellaneous charges include all items not reported under any other category, such as transportation to and from medical service providers (after discharge from rehabilitation), appropriate long-distance phone calls, and care and maintenance of helper animals (excluding pets).

### 3.4. Estimation of the Incidence of TAAAs in the United States

While the exact epidemiology remains unknown, the incidence of TAAAs may be estimated based on larger studies on Thoracic Aortic Aneurysms, of which TAAAs comprise approximately 6% (10). An early study on a Midwestern community in the United States calculated an incidence of approximately 6 thoracic aneurysms per 100,000 person-years (11). Given that the U.S. population reaches 311034360 (March 2011) according to the U.S. Census Bureau, it may be estimated that 1120 Americans are yearly affected by TAAAs.

## 4. Results

### 4.1. Calculations of Nationwide Health Care Charges and Costs

Based on the estimations in section 3, the nationwide health care charges and costs for TAAA endovascular and surgical repair can be calculated. Given that:

- 100 US patients undergo types II and III TAAA endovascular repair (5),
- the Inpatient Hospital Charges (amount hospital billed for services) is $124698,
- 11 patients experience SCI,
- the average expenses per patient after paraplegia for the first year are $463116,
- the average expenses per patient after paraplegia for each subsequent year is $61550,
- the life-expectancy per patient aged 69 at injury and surviving at least one year post-SCI is 10.2.

We could estimate that the total yearly inpatient hospital charges in the US are $12484324, the total average expense for patients affected by paraplegia during the first 12-month after endovascular repair is $4882291, and the total average expense after 10 years for ER patients affected by paraplegia is $6316183. For the counterpart, given that:

- 319 undergo types II and III TAAA surgical repair (5),
- the inpatient hospital charges (amount hospital billed for services) is $117985,
- 50 patients experience SCI,
-the average expenses per patient after paraplegia for the first year are $463116,
- the average expenses per patient after paraplegia for each subsequent year is $61550,
- the life-expectancy per patient aged 69 at injury and surviving at least 1 year post-SCI is 10.2.

We could estimate that the total yearly inpatient hospital charges in the US are $37612665, the total average expense for patients affected by paraplegia during the first 12-month after surgical repair is $23179110, and the total average expense after 10 years for SR patients affected by paraplegia is $33421910.

### 4.2. Calculation of Mortality Due to Types II and III TAAAs in the US

Based on the relevant figures presented above, we could calculate the nationwide mortality for patients who underwent types II and III TAAA endovascular and surgical repair. For 100 US patients undergoing types II and III TAAA endovascular repair yearly, the 30-day mortality is 8, and the 1-year mortality is 32. For 319 US patients undergoing types II and III TAAA surgical repair yearly, the 30-day mortality is 47 and the one-year mortality is 68. Hence, 55 patients with a type II or III TAAA would die after 30 days, and 100 would after one year. The potential risk of TAAA rupture for untreated patients may also be estimated. Between 1958 and 1985 in Malmö, Sweden, which has stable urban population and an autopsy prevalence of 83%, TAAs were found in 489 per 100,000 autopsies in men and 437 per 100,000 autopsies in women (12), which corresponds to 18 per 100,000 autopsies per year in men and 16 per 100,000 autopsies per year in women. When applying these figures to the U.S. population (13), we found that 1637 Americans are autopsied yearly with a type II or III TAAA, as depicted in Table 4. 

**Table 4.** First Year Post Injury Mean Charges Incurred by Persons With Paraplegia in 2009

Injury	Charges, $^a^
**Acute care**	256992
**Rehabilitation**	133300
**Rehospitalizations**	25533
**Nursing home care**	1103
**Outpatient services**	3913
**Physician fees**	838
**Durable equipment**	5368
**Environmental modifications**	9442
**Medications**	1356
**Supplies**	2003
**Attendant care**	21168
**Vocational rehabilitation**	891
**Miscellaneous charges**	1209
**First Year charges**	463116

^a^ US Dollar

## 5. Discussion

The elimination of spinal cord injury and mortality in the United States via one type of endovascular repair with the same average costs as endovascular treatments would benefit both the economy and welfare of the country. As the current nationwide inpatient hospital charges for type II or III TAAA repair are $12484324 and $37,612,665 for endovascular repair and surgical repair respectively, and as the total average expenses for patients affected by paraplegia during the first 12-month after endovascular repair are $4882291 and $23179110 after surgical repair, the US economy would save at least $53189742 (4882291 + 23179110 + 37612665 - 12484324 = 53189742) after one year. Moreover, it may be assumed that 100 lives of operated patients would be saved after one year, and 1637 types II and III TAAA ruptures would be avoided yearly in the United States due to the use of such technique.

Moreover, in 2010 Gopaldas et al. (8) compared thoracic aortic aneurysm (TAA) outcomes between SR and ER in nearly 12000 patients using the US Nationwide Inpatient Sample (NIS) database, providing a comprehensive analysis of hospital charges for isolated descending TAAs. Hospital charge information captured in the NIS represents the hospitals billed amount for services, but does not reflect how much the hospital services actually cost or the specific amounts that the hospitals received in payment. We assumed that hospital charges for TAAAs were similar to those for descending TAAs. The Gopaldas et al. study found that ER was associated with significantly greater hospital charges. Indeed, though the unadjusted hospital charges were similar for the ER and SR patients ($116329 ± $109006 vs. $117985 ± $80823; P = 0.5), after risk adjustment using multivariable linear regression analysis, ER was associated with an additional hospital charge of $6713 (P < 01). It may therefore be estimated that hospital charges for the ER patients equal $124698 ± $109006 and $117985 ± $80823 for SR patients.

The incidence of SCI following TAAA repair, particularly in extensive type II TAAAs, may be as high as 22% for an SR and 19% for an ER (4). Spinal cord ischemia causes not only devastating and costly physical and social disability, but also reduced survival at follow-up (14). Accordingly, with advancing medical technology and increasing life expectancies, the direct costs of SCI are going up at a rapid pace. Besides, with exceedingly high federal budget deficits and public unwillingness to support tax increases, both the administration and Congress are vigorously searching for ways to reduce federal spending on a variety of social, health and welfare programs.

The 2011 DeVivo et al. article (15) provided a detailed, accurate and updated cost analysis of health care charges resulting from paraplegia in the United States. The methodology and the parameters needed to be considered in our calculations are as follows: The study population for these parameters included a random sample of 508 patients originally treated between 1973 and 1988 at a model system and enrolled in the NSCISC database (16, 17). Acute care charges were based on the experiences of all persons admitted to a model system within 24 hours of injury between 2000 and 2006 (n = 1676) who had complete data on these items (n = 1508). They included emergency room fees, charges for room and board, X-ray, laboratory, pharmacy, central supply, intensive care unit, operating room, recovery room, anesthesia, nuclear medicine, respiratory therapy, and other miscellaneous inpatient charges occurring prior to transfer to the rehabilitation service. Rehabilitation charges were based on the experiences of all persons admitted to a model system within 24 hours of injury between 2000 and 2006 (n = 1676) who received inpatient rehabilitation at the model system and had complete data on these items (n = 1599). They included all charges for room and board, X-ray, laboratory, pharmacy, central supply, nuclear medicine, rehabilitation medicine, respiratory therapy, physical therapy, occupational therapy, recreational therapy, speech therapy, psychological services, and other miscellaneous items occurring during inpatient rehabilitation.

There were seven limitations for this study.

1. We relied on studies with their own biases to provide an estimation of the potential economic impacts in the United States.
2. The evolution of ER devices to treat patients affected by extensive TAAAs occurred throughout the period of the present review and continues today.
3. We extrapolated local data to issue a nationwide estimation.
4. We assumed the mean hospital charges of an ER eliminating mortality and paraplegia were equivalent to the mean hospital charges of endovascular repairs.
5. We assumed that hospital charges for TAAAs were similar to those for descending TAAs.
6. We assumed that the mortality rates for SR and ER patients with types II and III TAAAs were constant from 1 to 10 years after repair to calculate the total average expense after 10 years for SR and ER patients affected by paraplegia.
7. More empirical and clinical data outcomes would be needed to justify cost calculations.
